# Identification and Validation of Autophagy-Related Genes in Diabetic Retinopathy

**DOI:** 10.3389/fendo.2022.867600

**Published:** 2022-04-29

**Authors:** Nan Wang, Linfeng Wei, Die Liu, Quyan Zhang, Xiaobo Xia, Lexi Ding, Siqi Xiong

**Affiliations:** ^1^ Eye Center of Xiangya Hospital, Central South University, Changsha, China; ^2^ Hunan Key Laboratory of Opthalmology, Central South University, Changsha, China; ^3^ Department of General Surgery, Zhongshan Hospital of Dalian University, Dalian, China

**Keywords:** diabetic retinopathy, autophagy, differentially expressed genes, protein-protein interaction network, MAPK3

## Abstract

**Background:**

Diabetic retinopathy (DR) is one of the most common microvascular complications of diabetes, which is associated with damage of blood-retinal barrier and ischemia of retinal vasculature. It devastates visual acuity due to leakage of retinal vessels and aberrant pathological angiogenesis in diabetic patients. The etiology of DR is complex, accumulated studies have shown that autophagy plays an important role in the pathogenesis of DR, but its specific mechanism needs to be further studied.

**Methods:**

This study chose the online Gene Expression Omnibus (GEO) microarray expression profiling dataset GSE146615 to carry on the research. Autophagy-related genes that were potentially differentially expressed in DR were screened by R software. Then, the differentially expressed autophagy-related genes were analyzed by correlation analysis, tissue-specific gene expression, gene-ontology (GO) enrichment analysis, Kyoto Encyclopedia of Genes and Genomes (KEGG) pathway enrichment analysis and protein-protein interaction (PPI) network analysis. Finally, retinal pigment epithelial cell line (ARPE-19) incubated with high glucose (HG) was used to mimic the DR model, and the mRNA level of key genes was verified by quantitative real-time polymerase chain reaction (qRT-PCR) *in vitro*.

**Results:**

A total of 23 differentially expressed autophagy-related genes (9 up-regulated genes and 14 down-regulated genes) were identified by differential expression analysis. The analysis of tissue-specific gene expression showed that these differentially expressed autophagy-related genes were enriched in the retina. GO and KEGG enrichment analysis showed that differentially expressed autophagy-related genes were significantly enriched in autophagy-related pathways such as regulation of autophagy and macroautophagy. Then 10 hub genes were identified by PPI network analysis and construction of key modules. Finally, qRT-PCR confirmed that the expression of MAPK3 in the DR model was consistent with the results of bioinformatics analysis of mRNA chip.

**Conclusion:**

Through bioinformatics analysis, we identified 23 potential DR autophagy-related genes, among which the down-regulated expression of MAPK3 may affect the occurrence and development of DR by regulating autophagy. It provides a novel insight into the pathogenesis of DR.

## Highlights

• Differentially expressed genes (DEGs) related to autophagy in DR patients were identified.
• Major enrichment pathways of autophagy-related differential genes identified by bioinformatics, and the top 10 hub genes were identified.
• Experimental validation showed that down-regulation of MAPK3 gene might associated with DR by regulating autophagy.

## Introduction

Diabetic retinopathy (DR) is one of the most common and harmful microvascular complications of diabetes, and it is also a common eye disease that causes blindness (1). Most patients progress into DR after 20 years of diabetes (2, 3), and about half of the patients with untreated proliferative retinopathy will go blind within 5 years (4). It imposes a heavy economic burden on the families, health systems and societies. Previous studies have shown that oxidative stress, endoplasmic reticulum stress, apoptosis and autophagy (5–8) can induce mild and chronic retinal inflammation in retinal tissues (9), resulting in retinal vessel hyperpermeability (10), retinal angiogenesis and retinal neuron injury (11).

Autophagy is a process in which autophagy engulfs its own cytoplasmic proteins or organelles and encapsulates them into vesicles, fuses with lysosomes to form autophagy lysosomes, and degrades the contents of autophagy. It meets the demand of cellular metabolic needs and is involved in the renewal of some organelles. It is highly conserved in evolution and crucial for the degradation and circulation of cellular substances (12). Autophagy disorders may have fatal consequences to the cells and result in some ocular diseases (13), such as age-associated macular degeneration (AMD), glaucoma and other eye diseases (14–21). For example, glucosamine (GlcN) can induce autophagy to reduce photoreceptor outer segment (POS)-derived lipofuscin-like autofluorescence (LLAF) in retinal pigment epithelial (RPE) cells through the AMPK-mTOR pathway, which provides a novel insight for AMD (19). In the glaucoma model, the neurosteroid allopregnanolone (AlloP) can reduce the apoptosis of retinal ganglion cells (RGC) by activating autophagy (21).

**Figure 1:**
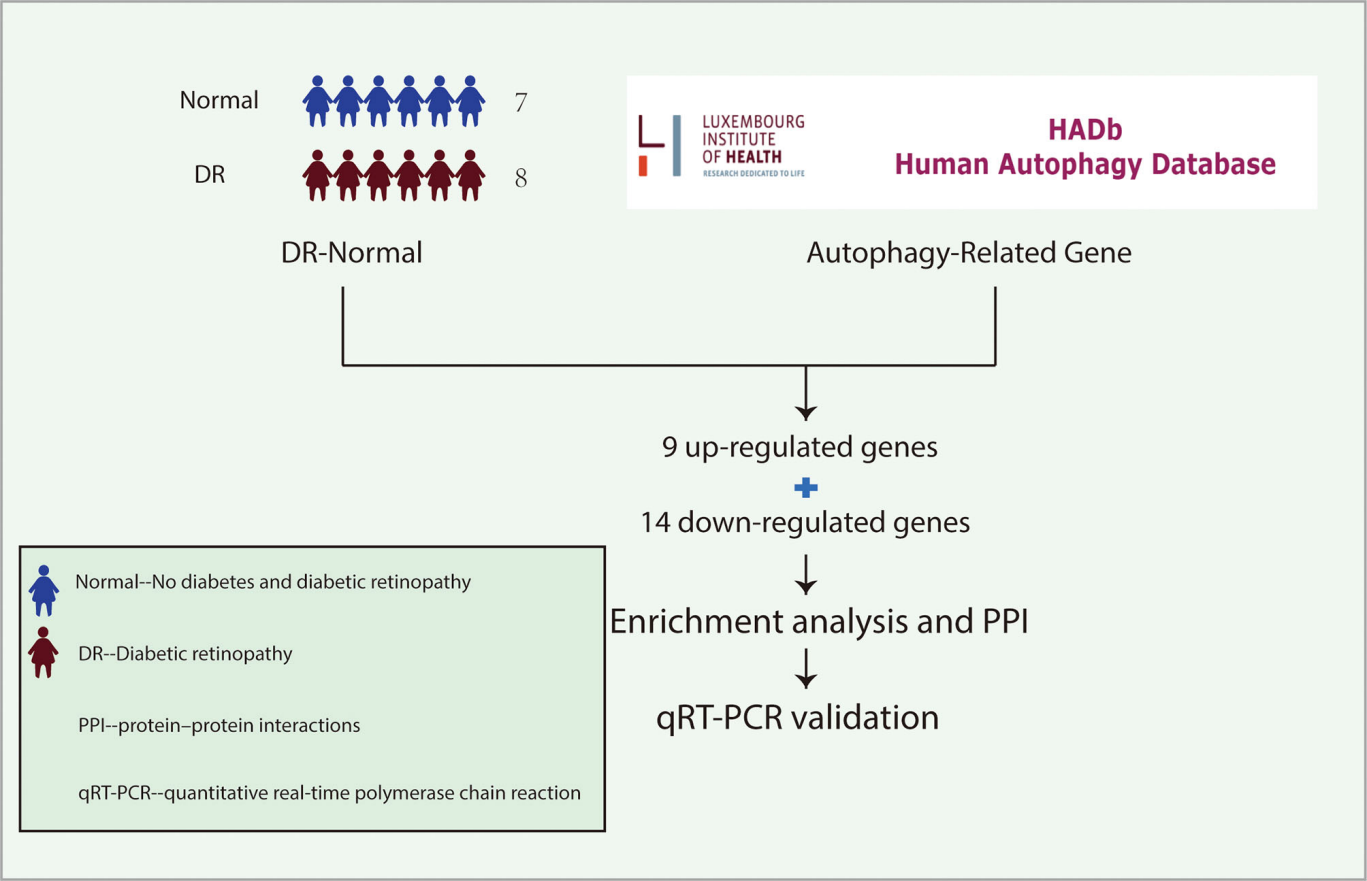
The idea of experimental design. The gene expression profiles of LCLs extracted from the peripheral blood of 7 non-diabetic individuals and 8 patients with DR were cultured under SG and HG conditions respectively in the GSE146615 dataset. 232 autophagy-related genes were collected from The Human Autophagy Database. Then, 9 up-regulated genes and 14 down-regulated genes were screened by differential analysis. After enrichment analysis and PPI network construction, 10 hub genes were identified. Finally, qRT-PCR was used to verify *in vitro* DR model. LCLs, lymphoblastoid cell lines; DR, diabetic retinopathy; SG, standard glucose; HG, high glucose; PPI, protein-protein interaction; qRT-PCR, quantitative real-time polymerase chain reaction.

Recently, the role of autophagy in DR has been gradually uncovered. Damage of outer blood-retina barrier due to diabetes is key to the pathogenesis of diabetic macular edema. It leads to decrease of visual acuity in patients with DR. RPE cells are the main components of the outer blood-retina barrier. It has been demonstrated that the outer blood-retina barrier was destroyed in diabetes by modulation of autophagy in RPE cells. However, the underlying mechanism of autophagy in devastating RPE cells under diabetes stress is still unclear. In this study, we analyzed the previously published dataset containing samples from DR and non-diabetic individuals to identify the differentially expressed genes (DEGs) related to DR. It was further analyzed to figure out the correlation between the differentially expressed autophagy-related genes in DR. Then, functional enrichment and protein-protein interaction (PPI) network analysis were used to clarify the interaction and biological function of these genes. 10 hub genes were identified by PPI network analysis, and it was further verified in the DR model by quantitative real-time polymerase chain reaction (qRT-PCR) *in vitro*. We found that the expression of MAPK3 was consistent with the results of bioinformatics analysis of mRNA chip in the DR model, suggesting it was involved in the development of DR by regulating autophagy (**Figure 1**).

## Materials And Methods

### Microarray Data and Autophagy-Related Genes Datasets

The mRNA expression profile dataset GSE146615 was downloaded from the Gene Expression Omnibus (GEO) database (http://www.ncbi.nlm.nih.gov/geo/). 232 autophagy-related genes were obtained from The Human Autophagy Database (http://www.autophagy.lu/index.html). GSE146615 was in GPL10558 platform. The dataset included 7 individuals without diabetes, 7 patients with type 1 diabetes (T1D) without complications from DR and 8 patients with DR. Lymphoblastoid cell lines (LCLs) were extracted from the peripheral blood of 22 individuals and processed with standard glucose (SG) and high glucose (HG), respectively. In this study, the data of LCLs of 7 non-diabetic individuals treated with SG and LCLs of 8 patients with DR treated with HG were extracted for follow-up analysis.

### Differential Expression Analysis of Autophagy-Related Genes

The DEGs related to autophagy were screened by the “limma” package in R software. The genes with P value < 0.05 were considered to be DEGs. Then, the “heatmap” and “ggplot2” packages in R software are used to draw heatmap and volcano plot and box plot respectively to visualize the differential genes.

### Correlation Analysis of DEGs and Tissue-Specific Gene Expression Analysis

The correlation analysis of differentially expressed autophagy-related genes was carried out by using Spearman correlation in the “corrplot” package of R software. The tissue-specific expression of differentially expressed autophagy-related genes was analyzed on the BioGPS website (http://biogps.org).

### GO and KEGG Pathway Enrichment Analysis of Differentially Expressed Autophagy-Related Genes

We used the “GO plot” package in R software to analyze the functional enrichment of differentially expressed autophagy-related genes, including Gene Ontology (GO) terms and Kyoto Encyclopedia of Genes and Genomes (KEGG) pathways. In the GO analysis, we evaluated the enriched biological processes (BPs), molecular functions (MFs) and cellular components (CCs).

### PPI Network

The STRING database (https://string-db.org/) of known and predicted protein-protein interactions was used to analyze the PPI network of differentially expressed autophagy-related genes, and then the Cytoscape v3.9.0 software was used to visualize and construct the PPI network, and finally 10 hub genes were identified by cytoHubba plugin.

### Cell Culture and Cell Grouping

Human retinal pigment epithelial cell line (ARPE-19) was purchased from the Procell Life Science & Technology in China (CL-0026) and cultured in DMEM/F12 medium containing 10% fetal bovine serum (FBS) (Gibco, USA) and 1% Antibiotic-Antimycotic (Gibco, USA) at 37°C with 5% carbon dioxide. When the cell density reached 80%, it was washed with PBS (Gibco, USA) and treated with 0.05% trypsin (Gibco, USA) for passage at the proportion of 1:3.

The logarithmic growth phase cells with good growth condition were inserted into 6-well plates with 1.2×10^6^ cells per well. The cells were divided into two groups: the HG treated group and the normal group. The HG treated group was cultured in the medium containing 30mmol/L D-glucose, and the normal group was cultured in the SG medium. The 2.5mL medium was added to each well and cultured at 37°C for 48 hours.

### RNA Extraction and qRT-PCR

RNA was extracted from ARPE-19 cells using TRIzol kit (Invitrogen, USA) according to the manufacturer’s plan, and 2 × SYBR Green qPCR Master Mix and UEIris II RT-PCR System using a First-Strand cDNA, as well as Synthesis Kit (Suzhou Yuheng Biological Co., Ltd.) were used for reverse transcription and qRT-PCR. The primers were designed and synthesized by Sangon Biotech Co., Ltd (Shanghai, China), and the sequence was listed in **Supplementary Table S1**. Configure the reaction mixture according to the system in **Supplementary Table S2**, gently swirl and centrifuge the reaction mixture, transfer it to the PCR plate, and carry out the experimental reaction according to the procedure in **Supplementary Table S3**. Finally, the results were analyzed by real-time PCR detection system (ABI). The expression level of mRNA was calculated by 2^−ΔΔCt^ method, and the relative expression level of gene mRNA was normalized by β-actin. Sterilized deionized water was used instead of nucleic acid template as negative control to ensure the quality of primers and no pollution of the system.

### Statistical Analysis

All the experimental data were statistically analyzed by GraphpadPrism software (version3.6.2), and 3 independent experiments were carried out. The gene expression level of the sample was compared by Student’s t-test, and the difference was considered to be statistically significant when P < 0.05.

## Results

### Identification of Differentially Expressed Autophagy-Related Genes

**Figure 2:**
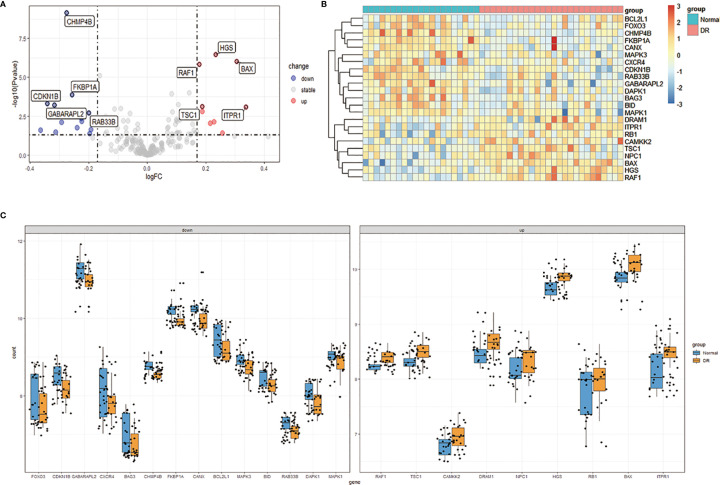
Differentially expressed autophagy-related genes in DR patients (DR group) and non-diabetic individuals (Normal group). **(A)**, Volcano plot of 232 differentially expressed autophagy-related genes. The red dots in the picture represent significantly up-regulated genes, blue dots represent significantly down-regulated genes, black dots represent genes that are not differentially expressed, and the five genes that are most significantly up-regulated or down-regulated are marked. **(B)**, The heatmap of 232 differentially expressed autophagy-related genes. Red represents up-regulated genes and blue represents downregulated genes. **(C)**, The boxplot of 23 differentially expressed autophagy-related genes in DR and normal samples. It includes 9 up-regulated genes and 14 down-regulated genes. DR, diabetic retinopathy.

We downloaded the Expression profiling by array dataset GSE146615 from the GEO database, and selected LCLs cultured in SG from 7 non-diabetic individuals (Normal group) and 8 patients with T1D and proliferative diabetic retinopathy (PDR) cultured in HG (DR group). Next, we analyzed the expression of 232 autophagy-related genes in the sample by R software, identified 9 up-regulated genes and 14 down-regulated genes, and then plotted 23 differentially expressed autophagy-related genes between the DR group and the normal group (**Figures 2A, B**). In addition, the box chart showed the expression patterns of 23 autophagy-related genes differentially expressed between DR patients and normal samples (**Figure 2C**). The first five up-regulated genes include HGS, BAX, RAF1, TSC1 and ITPR1, and the first five down-regulated genes include CHMP4B, FKBP1A, CDKN1B, GABARAPL2 and RAB33B (**Table 1**).

**Table 1:** The 23 differentially expressed autophagy-related genes in DR samples compared to healthy samples.

Gene Symbol	logFC	Changes	P-value	Adj. P-value	probe_id
HGS	0.2329999	Up	3.340629E-07	3.965281E-05	ILMN_1715994
BAX	0.3046402	Up	9.690973E-07	9.408659E-05	ILMN_2321064
RAF1	0.1775394	Up	1.512913E-06	1.344553E-04	ILMN_1813489
TSC1	0.1869165	Up	7.643098E-04	1.695813E-02	ILMN_2246510
ITPR1	0.3363179	Up	7.942211E-04	1.751126E-02	ILMN_1789505
CAMKK2	0.1882823	Up	1.550804E-03	2.798067E-02	ILMN_1743021
NPC1	0.2273093	Up	7.180077E-03	7.976330E-02	ILMN_1713505
DRAM1	0.2153663	Up	8.943841E-03	9.250777E-02	ILMN_1669376
RB1	0.2570772	Up	3.879564E-02	2.349566E-01	ILMN_1696591
CHMP4B	-0.2763448	Down	7.283285E-10	3.658531E-07	ILMN_1771233
FKBP1A	-0.2572678	Down	1.309686E-04	4.539371E-03	ILMN_1757072
CDKN1B	-0.3413209	Down	5.099296E-04	1.251711E-02	IILMN_2196347
GABARAPL2	-0.3170168	Down	6.248193E-04	1.457351E-03	ILMN_1796458
RAB33B	-0.1992503	Down	2.001880E-03	3.376344E-02	ILMN_1727738
CANX	-0.2521261	Down	3.244758E-03	4.777541E-02	ILMN_2401057
MAPK3	-0.2250177	Down	4.241640E-03	5.711548E-02	ILMN_1667260
BID	-0.2237670	Down	6.619973E-03	7.562566E-02	ILMN_1763386
BAG3	-0.2929996	Down	7790892E-03	8451716E-02	ILMN_1659766
BCL2L1	-0.2402096	Down	1.767939E-02	1.446571E-01	ILMN_1654118
MAPK1	-0.1921206	Down	2.328004E-02	1.711489E-01	ILMN_2235283
FOXO3	-0.3645971	Down	2.510350E-02	1.798444E-01	ILMN_1681703
CXCR4	-0.3131656	Down	3.347568E-02	2.145289E-01	ILMN_1801584
DAPK1	-0.1962781	Down	4.186049E-02	2.453443E-01	ILMN_1708340

### Correlation and Tissue-Specific Expression of Differentially Expressed Autophagy-Related Genes

**Figure 3:**
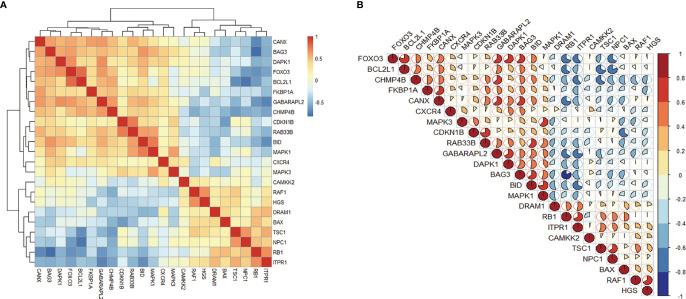
Correlation analysis of 23 differentially expressed autophagy-related genes. **(A, B)**, Correlation heatmap.

In order to explore the expression correlation of these 23 autophagy-related genes, the correlation analysis has been performed by bioinformatics methods. The results showed that there was a high correlation between up-regulated genes and down-regulated genes, respectively (**Figures 3A, B**). At the same time, we identified the expression of these 23 genes in human retina by BioGPS. Except for CAMKK2, the expression levels of the other 22 genes in the retina were higher than the average levels in the tissues or organ systems of the whole body. Among them, the expression levels of 6 genes such as RAF1 in the human retina were more than three times the median, indicating that these autophagy-related genes were enriched in the human retina (**Table 2**).

**Table 2:** Expression levels of dierentially expressed genes identified by BioGPS in retinal tissues.

Gene	Expression level	Median	Gene	Expression level	Median
>3×M					
RAF1	258.83±49.09	82.7	CDKN1B	456.62±99.99	120.3
ITPR1	86.50±26.05	11	GABARAPL2	2019.33±343.13	551.6
			BAG3	86.45±2.13	23.5
			CXCR4	25.30±2.50	4.3
>1×M			CHMP4B	687.08±74.31	376.3
HGS	130.57±16.11	63.5	FKBP1A	68.02±3.06	31.3
BAX	7.55±0.475	6.4	RAB33B	23.38±2.89	16.4
TSC1	174.60±24.85	92.5	CANX	960.05±86.58	458.4
NPC1	7.75±0.375	6.43	MAPK3	152.32±45.69	77
DRAM1	4.50±0.300	3.9	BID	11.25±0.675	10.2
RB1	26.77±1.36	20.3	BCL2L1	15.18±0.590	10.2
			MAPK1	21.25±0.725	15.5
			FOXO3	12.50±0.250	4.7
			DAPK1	9.20±0.500	7.9
<1×M					
CAMKK2	27.10±10.80				

### Functional and Pathway Enrichment of The Differentially Expressed Autophagy-Related Genes

**Figure 4:**
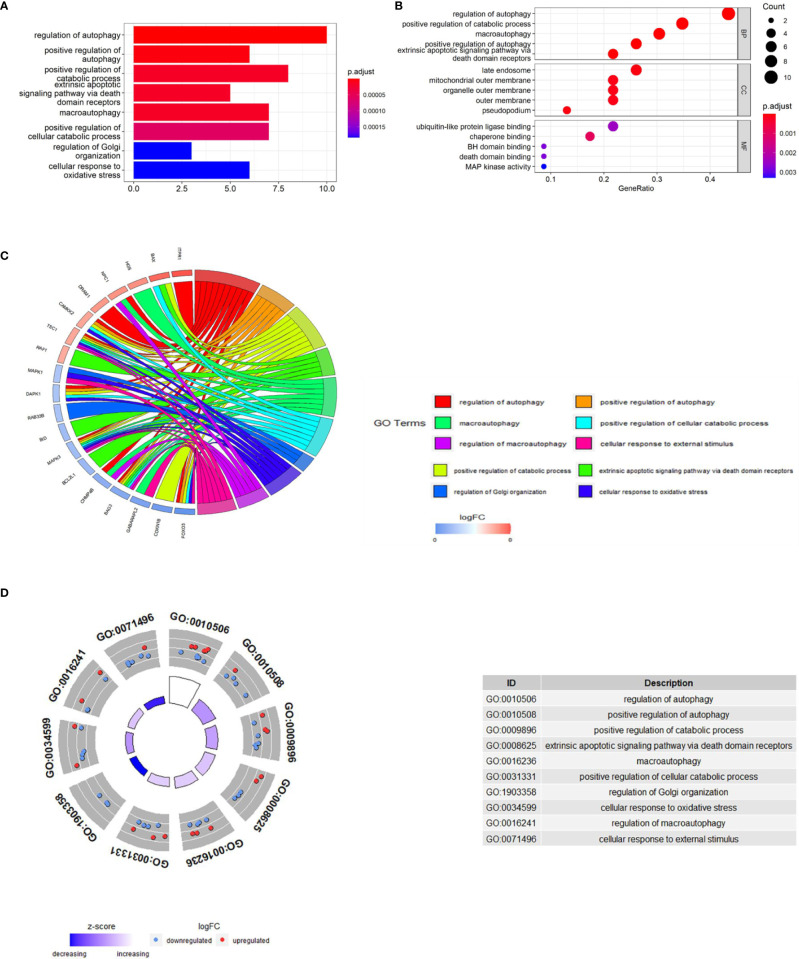
GO enrichment analysis of 23 differentially expressed autophagy-related genes, including BPs, CCs and MFs. **(A)**, Bar plot of enriched GO terms. **(B)**, Bubble plot of enriched GO terms. **(C)**, Chordal graph of enriched GO terms. It shows the relationship between DEGs and the first 10 enriched GO pathways. **(D)**, Eight Diagrams of enriched GO terms. GO, Gene Ontology; BPs, biological processes; CCs, cellular components; MFs, molecular functions; DEGs, differentially expressed genes.

**Figure 5:**
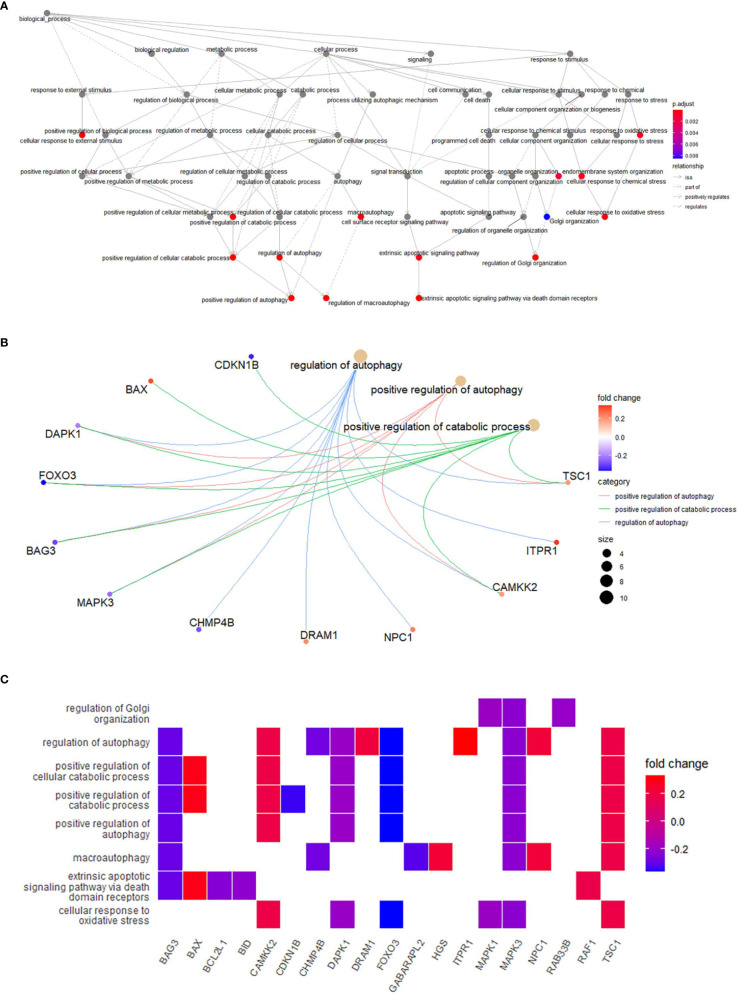
**(A)**, Relationships between enriched pathways. **(B)**, Common genes in the most top pathways. **(C)**, Heatmap-like functional classification.

**Figure 6:**
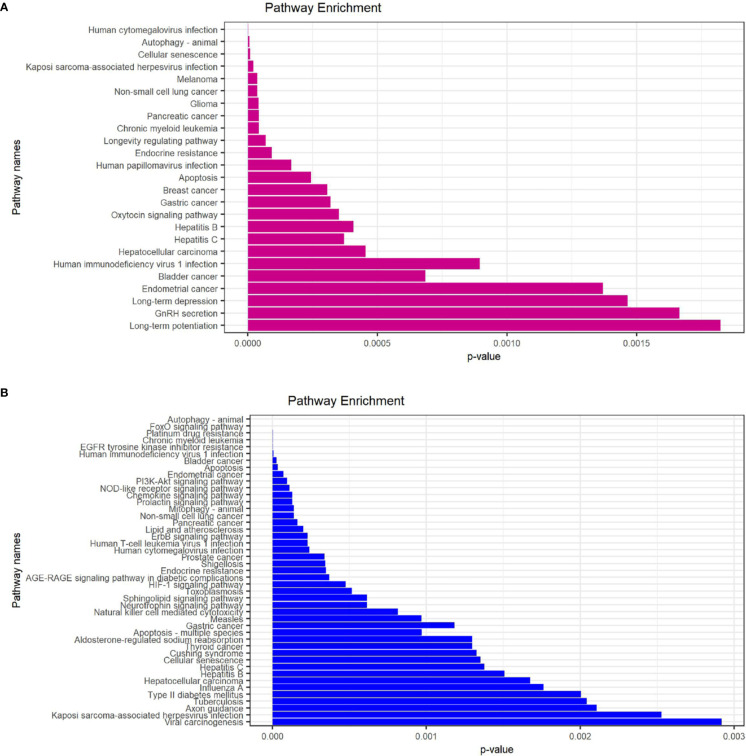
KEGG enrichment analysis of 23 differentially expressed autophagy-related genes. **(A)**, KEGG analysis of 9 up-regulated expressed autophagy-related genes. **(B)**, KEGG analysis of 14 down-regulated expressed autophagy-related genes. KEGG, Kyoto Encyclopedia of Genes and Genomes.

In order to explore the potential function of differential genes more deeply from the level of biological function, we used R software for GO and KEGG enrichment analysis (**Table 3**). GO enrichment analysis showed that the differential genes were significantly enriched in 441 BPs, 20 MFs and 19 CCs. Among them, the most prominent projects involved regulation of autophagy, positive regulation of catabolic process, macroautophagy, positive regulation of autophagy (BPs); late endosome, mitochondrial outer membrane, organelle outer membrane (CCs); ubiquitin-like protein ligase binding, chaperone binding, BH domain binding (MFs) (**Figures 4A–D**). The relationship between these pathways was shown in the (**Figure 5A**). There were 12 common genes in the three most prominent pathways, namely CDKN1B, BAX, DAPK1, FOXO3, BAG3, MAPK3, CHMP4B, DRAM1, NPC1, CAMKK2, ITPR1 and TSC1 (**Figure 5B**). Besides, we analyzed the expression of differential genes in the significantly enriched pathway and showed the results in the Heatmap-like functional classification map (**Figure 5C**). In addition, the KEGG results showed that the enrichment was mainly in the process of autophagy, human cytomegalovirus infection and so on (**Figures 6A, B**).

**Table 3:** Functional and pathway enrichment analyses for module genes.

Term	Description	Count	P-value	Adj. P-value	Genes
Biological processes					
GO:0010506	Regulation of autophagy	10	2.07E-12	2.88E-09	TSC1, ITPR1, CAMKK2, NPC1, DRAM1, CHMP4B, MAPK3, BAG3, FOXO3, DAPK1
GO:0010508	Positive regulation of autophagy	6	5.40E-09	3.76E-06	TSC1, CAMKK2, MAPK3, BAG3, FOXO3, DAPK1
GO:0009896	Positive regulation of catabolic process	8	3.53E-08	1.64E-05	
Cellular component					
GO:0005770	Late endosome	6	6.11E-07	7.78E-05	HGS, NPC1, CHMP4B, MAPK3, MAPK1, CXCR4
GO:0005741	Mitochondrial outer membrane	5	2.75E-06	1.24E-04	BAX, RAF1, BID, BCL2L1, FOXO3
GO:0031968	Organelle outer membrane	5	4.96E-06	1.40E-04	
Molecular functions					
GO:0051087	Chaperone binding	4	7.08E-06	9.56E-04	BAX, TSC1, CDKN1B, BAG3
GO:0044389	Ubiquitin-like protein ligase binding	5	3.61E-05	2.44E-03	HGS, RB1, GABARAPL2, BID, CXCR4
GO:0004674	Protein serine/threonine kinase activity	5	1.64E-04	3.29E-03	RAF1, CAMKK2, MAPK3, MAPK1, DAPK1
KEGG pathway					
hsa04140	Autophagy – animal	6	2.11E-08	3.12E-06	GABARAPL2, RAB33B, MAPK3, BCL2L1, MAPK1, DAPK1
hsa04068	FoxO signaling pathway	5	7.37E-07	5.46E-05	CDKN1B, GABARAPL2, MAPK3, MAPK1, FOXO3
hsa05163	Human cytomegalovirus infection	5	8.23E-07	1.12E-04	BAX, RAF1, TSC1, ITPR1, RB1

The top 3 terms were selected based upon Adj. P-value rankings when>3 terms were enriched for a given category.

### PPI Network Analysis and Hub Gene Identification

**Figure 7:**
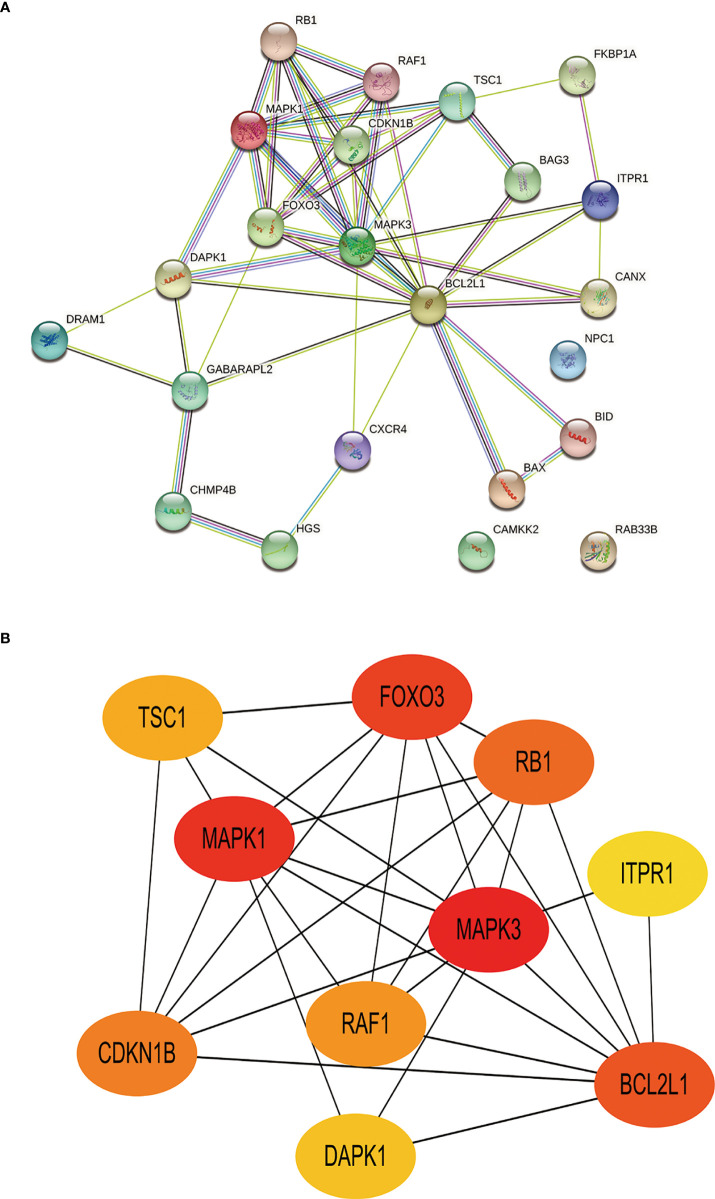
Construction of PPI network and identification of hub genes. **(A)**, The PPI between 23 differentially expressed autophagy-related genes was constructed by using the STRING database. The node represents the gene, and the edge represents the relationship between the genes. **(B)**, The top 10 key genes were screened through the PPI network map. Different colors in the image only represent different genes and have no other substantive meaning. PPI, protein-protein interaction.

Toward a deeper understanding of the interaction between differentially expressed autophagy-related genes, we introduced these genes into the Search Tool for the Retrieval of Interacting Genes (STRING) to construct a PPI network (**Figure 7A**). The first 10 hub genes with the highest value were screened by Cytoscape (v3.9.0) (**Table 4**). Among them, TSC1, RAF1, RB1, ITPR1 is up-regulated and CDKN1B, MAPK1, FOXO3, DAPK1, MAPK3, BCL2L1 was down-regulated. The disorders of these autophagy motifs may be closely related to the occurrence and development of DR (**Figure 7B**).

**Table 4:** Top 10 in network ranked by MCC method.

Rank	Gene ID	Gene name	Score	Changes
1	MAPK3	Mitogen-Activated Protein Kinase 3	278	Down
2	MAPK1	Mitogen-Activated Protein Kinase 1	270	Down
3	FOXO3	Forkhead Box O3	266	Down
4	BCL2L1	BCL2-like 1	261	Down
5	RB1	Retinoblastoma 1	240	Up
6	CDKN1	Cyclin-Dependent Kinase Inhibitor 1B Raf-1	144	Down
7	B RAF1	Proto-Oncogene	120	Up
8	TSC1	Tuberous Sclerosis 1	26	Up
9	DAPK1	Death-Associated Protein Kinase 1	10	Down
10	ITPR1	Inositol 1,4,5-Triphosphate Receptor, Type 1	7	Up

### Validation the Differentially Expressed Autophagy-Related Genes in Diabetic Model

**Figure 8:**
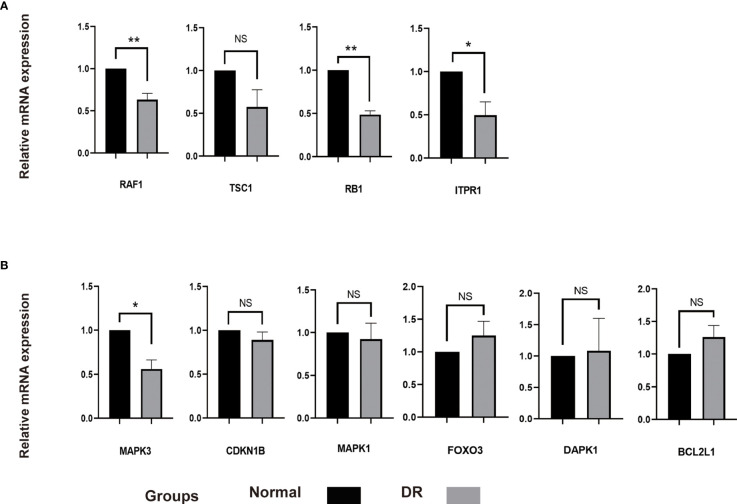
The mRNA level of 10 hub genes were measured in ARPE-19 cells. **(A)**, The mRNA level of RAF1, TSC1, RB1 and ITPR1 were evaluated in cell samples by qRT-PCR. **(B)**, The mRNA level of MAPK3, CDKN1B, MAPK1, FOXO3, DAPK1 and BCL2L1 were measured in cell samples by qRT-PCR. P-values were calculated using a two-sided unpaired Student’s t-test. *P < 0.05; **P < 0.01; ns, non-significant. ARPE-19, retinal pigment epithelial cell line; qRT-PCR, quantitative real-time polymerase chain reaction.

It was found that autophagy in RPE cells was related to damage of outer blood retinal barrier in diabetic retinopathy. Meanwhile, autolysosomes and autophagy associated markers were increased in RPE cells under HG condition (22–24). In this study, we incubated ARPE-19 cells with HG (30mM) to simulate an DR model *in vitro*. Interestingly, the expression of RAF1, RB1 and TPR1 were decreased and the level of TSC1 remained unchanged in HG treated ARPE-19 cells compared with the normal control cells. These genes were predicted to be upregulated according to aforementioned bioinformatics analysis. The level of CDKN1B, MAPK1, FOXO3, DAPK1 and BCL2L1 expected to be downregulated were found to be similar in both SG and HG treated ARPE-19 cells. The level of MAPK3 was decreased in ARPE-19 cells under HG condition, indicating MAPK3 and downstream signaling pathway might participate in the progression of DR by regulating autophagy in RPE cells (**Figure 8**).

## Discussion

DR is a chronic progressive complication of patients with diabetes, which is an important cause of blindness in patients with diabetes (1, 25, 26). The pathogenesis of DR is complex. Current studies have shown that a variety of metabolic pathways are involved in the formation of DR, such as the increase of polyol pathway, the activation of protein kinase C, oxidative stress and endoplasmic reticulum stress (5, 6). The abnormality of these pathways can not only cause microvascular complications such as the destruction of blood-retinal barrier (27, 28), but also lead to neurodegeneration and neuroinflammation (29). However, the exact pathogenic mechanism of DR has not been fully elucidated. Accumulating evidence show that autophagy, as the main catabolic pathway for the degradation and recycling of damaged proteins or organelles, may be involved in the pathogenesis of DR. Long-term hyperglycemia can cause autophagy disorder by inhibiting mTOR, resulting in the loss of retinal ganglion cells (30). In addition, low glucose (15mM) could induce mitochondrial autophagy in RPE cells, while under the stimulation of HG (50mM) or hydrogen peroxide, ROS could mediate the inactivation of mitochondrial autophagy-related proteins PINK1 and Parkin, and inhibited the occurrence of mitochondrial autophagy, indicating that glucose affected the occurrence of mitochondrial autophagy in RPE cells in a dose-dependent manner (31). Other studies have shown that knockout of high mobility group box1 (HMGB1) gene in RPE cells in the early stage of DR could save lysosome membrane permeabilization (LMP) through cathepsin B (CTSB)-dependent pathway. It restored the degradation ability of autophagy and thus protected RPE cells from apoptosis (32). The above results show that many forms of autophagy participate in the occurrence and development of DR, but its specific mechanism remains unclear. Further studies are required to broaden our knowledge of autophagy in the pathogenesis of DR.

In this study, we identified 23 potential autophagy-related genes in DR for the first time through bioinformatics analysis. GO and KEGG enrichment analysis showed that these genes were closely related to regulation of autophagy, positive regulation of catabolic process, macroautophagy and other signal pathways. Next, we further identified 10 hub genes related to DR, including TSC1, RAF1, RB1, ITPR1, CDKN1B, MAPK1, FOXO3, DAPK1, MAPK3 and BCL2L1 by using PPI network and key module analysis. The function of these genes in the occurrence of DR has been extensively studied. For example, Ras/Raf-1/MEK/ERK signal cascade can promote the activation of MMP9, and Raf kinase can also interact with VEGF to promote the loss of retinal capillary cells, which eventually leads to the development of DR (33–36). Overexpression of Raf-1 Kinase Inhibitory Protein (RKIP) can prevent the occurrence of diabetic retinal neurodegeneration by inhibiting p38-MAPK pathway (37). Hu-zhang-qing-mai-yin (HZQMY) can regulate P38 and NF-κB pathways by targeting MAPK3 and inhibit the release of ROS under HG exposure in a dose-dependent manner, thus inhibiting the proliferation of human retinal capillary endothelial cells (HRCECs) and having a certain effect on DR (38). Studies have shown that these autophagy-related genes regulate autophagy activity in tumor, cerebral ischemic stroke and osteoporosis (39–42). However, the role of these genes in modulating autophagy in DR has not been fully explored.

In current study, HG treated ARPE-19 cells were used as DR model to testify the function of potential autophagy-related genes. It is due to following reasons. Firstly, the dysfunction and loss of RPE cells has been found in diabetic model. It is associated with macular edema arising from diabetes-induced disruption of outer blood-retinal barrier. Therefore, RPE cells have been widely utilized as *in vitro* model for DR research (22). Secondly, both autophagy associated markers and autolysosomes are obviously detected in HG treated RPE cells, indicating this *in vitro* DR model is suitable for autophagy investigation (23, 24). Among 10 predicted DR-related hub genes, we showed that only the expression of MAPK3 was consistent with that of bioinformatics analysis of mRNA chip. We speculate that because the bioinformatics results were originated from peripheral blood lymphocytes of DR patients and non-diabetic individuals, the differences in cell type and culture condition may change gene expression and provide contradicting results. MAPK3 has been demonstrated to modulate autophagy by regulating mTOR pathway and Beclin-1 expression (43). Further investigation is required to elucidate the way by which MAPK3 control autophagy in DR models and cells.

## Conclusions

To sum up, 23 potential autophagy-related genes in DR were identified by bioinformatics analysis, 10 hub genes TSC1, RAF1, RB1, ITPR1, CDKN1B, MAPK1, FOXO3, DAPK1, MAPK3, BCL2L1 were identified by constructing PPI network and identifying key modules. MAPK3 was preliminarily identified by *in vitro* experiments, which may affect the occurrence and development of DR by regulating autophagy. In the future, further experiments are needed to investigate the regulatory role of MAPK3 in DR models in order to clarify its value as potential clinical biomarkers or therapeutic targets.

## Data Availability Statement

The datasets presented in this study can be found in online repositories. The names of the repository/repositories and accession number(s) can be found in the article/**Supplementary Material**.

## Author Contributions

NW analyzed the data. NW and LFW drafted the first draft. DL, QYZ, LXD, and XBX edited and provided comments to improve the manuscript. SQX designed this experiment and reviewed and revised the manuscripts. All authors contributed to the article and approved the final manuscript.

## Funding

This study was financially supported by The National Natural Science Foundation of China (No.81974137 to SQX), The National Natural Science Foundation of China (No.82070966 to LD), The Natural Science Foundation of Hunan Province (No.2019JJ40507 to XS), and the Science and Technology Innovation Program of Hunan Province (No.2021RC3026 to LD).

## Conflict of Interest

The authors declare that the research was conducted in the absence of any commercial or financial relationships that could be construed as a potential conflict of interest.

## Publisher’s Note

All claims expressed in this article are solely those of the authors and do not necessarily represent those of their affiliated organizations, or those of the publisher, the editors and the reviewers. Any product that may be evaluated in this article, or claim that may be made by its manufacturer, is not guaranteed or endorsed by the publisher.
