# Supplementary material for: Identification and Validation of Autophagy-Related Genes in Diabetic Retinopathy
Source: Front Endocrinol (Lausanne). 2022 Apr 29;13:867600. doi: 10.3389/fendo.2022.867600 (PMC9098829; doi:10.3389/fendo.2022.867600)
Supplement: Supplementary file 1 [file DataSheet_1.zip › Supplementary Table S1.DOCX]

| Gene | Primer Sequence (from 5' to 3')  Forword: 5'-CTCCACAGCCAGATCAGACA-3' Reverse: 5'-GCTGCCTGTTCAAGAACTCC-3' Forword: 5'-AGATGTCAAACGTGCGAGTG-3' Reverse: 5'-TCTCTGCAGTGCTTCTCCAA-3' Forword: 5'-CCACCCATATCTGGAGCAGT-3' Reverse: 5'-CAGTCCTCTGAGCCCTTGTC-3' Forword: 5'-GCAAGCACAGAGTTGGATGA-3' Reverse: 5'-CAGGTCGTCCATGAGGTTTT-3' Forword: 5'-ACCCATTCAGTTTCCAGTCG-3' Reverse: 5'-GCTACCAGCCTCTTCATTGC-3' Forword: 5'-ATGATCCCACGTCAATCCAT-3' Reverse: 5'-CCACCAGGACAACTTGGAGT-3' Forword: 5'-GGAAGCAACCCTCCTAAACC-3' Reverse: 5'-TTTCTGCTTTTGCATTCGTG-3' Forword: 5'-ACTATGACCCGACGGATGAG-3' Reverse: 5'-CTAACAGTCTGGCGGGAGAG-3' Forword: 5'-CTGATTCACCCACGAAGGTT-3' Reverse: 5'-TGCAAATCAGGTGCTTTCTG-3' Forword: 5'-TCTGGTCCCTTGCAGCTAGT-3' Reverse: 5'-CAGGGAGGCTAAGGGGTAAG-3' Forword: 5'-GGCATGGGTCAGAAGGATT-3' Reverse: 5'-TGGTGCCAGATTTTCTCCA-3' |
| --- | --- |
| TSC1  CDKN1B  MAPK1  FOXO3  RAF1  DAPK1  RB1  MAPK3  ITPR1  BCL2L1  β-Actin |  |
