# Supplementary material for: Identification and Validation of Autophagy-Related Genes in Diabetic Retinopathy
Source: Front Endocrinol (Lausanne). 2022 Apr 29;13:867600. doi: 10.3389/fendo.2022.867600 (PMC9098829; doi:10.3389/fendo.2022.867600)
Supplement: Supplementary file 1 [file DataSheet_1.zip › Supplementary Table S2.DOCX]

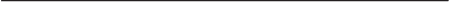
Reactive Components 20μL reaction system

Hieff qPCR SYBR Green Master Mix

Forward Primer （10 μM）

Reverse Primer （10 μM）

cDNA Template

Sterile Enzyme-free Water

10μL

0.4μL

0.4μL

1μL

8.2μL
