# Supplementary material for: Identification and Validation of Autophagy-Related Genes in Diabetic Retinopathy
Source: Front Endocrinol (Lausanne). 2022 Apr 29;13:867600. doi: 10.3389/fendo.2022.867600 (PMC9098829; doi:10.3389/fendo.2022.867600)
Supplement: Supplementary file 1 [file DataSheet_1.zip › Supplementary Table S3.DOCX]

Supplementary Table S3 | Amplification procedure.

| Cycle steps | Temperature | Time | Number of cycles | |
| --- | --- | --- | --- | --- |
| Predenaturation  Denaturation  Annealing / Extension  Melting curve stage | 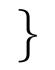5 min  95℃  95℃  60℃  10 sec  30sec  Instrument default settings | | | 1  40  1 |
